# Patterns of Multimorbidity in the Aged Population. Results from the KORA-Age Study

**DOI:** 10.1371/journal.pone.0030556

**Published:** 2012-01-23

**Authors:** Inge Kirchberger, Christa Meisinger, Margit Heier, Anja-Kerstin Zimmermann, Barbara Thorand, Christine S. Autenrieth, Annette Peters, Karl-Heinz Ladwig, Angela Döring

**Affiliations:** 1 Institute of Epidemiology II, Helmholtz Zentrum München, German Research Center for Environmental Health, Neuherberg, Germany; 2 MONICA/KORA Myocardial Infarction Registry, Central Hospital of Augsburg, Augsburg, Germany; 3 Institute of Epidemiology I, Helmholtz Zentrum München, German Research Center for Environmental Health, Neuherberg, Germany; Marienhospital Herne - University of Bochum, Germany

## Abstract

Multimorbidity is a common problem in aged populations with a wide range of individual and societal consequences. The objective of the study was to explore patterns of comorbidity and multimorbidity in an elderly population using different analytical approaches. Data were gathered from the population-based KORA-Age project, which included 4,127 persons aged 65–94 years living in the city of Augsburg and its two surrounding counties in Southern Germany. Information on the presence of 13 chronic conditions was collected in a standardized telephone interview and a self-administered questionnaire. Patterns of comorbidity and multimorbidity were analyzed using prevalence figures, logistic regression models and exploratory tetrachoric factor analysis. The prevalence of multimorbidity (≥2 diseases) was 58.6% in the total sample. Hypertension and diabetes (Odds Ratio [OR] 2.95, 99.58% confidence interval [CI] [2.19–3.96]), as well as hypertension and stroke (OR 2.00, 99.58% CI [1.26–3.16]) most often occurred in combination. This association was independent of age, sex and the presence of other conditions. Using factor analysis, we identified four patterns of multimorbidity: the first pattern includes cardiovascular and metabolic diseases, the second includes joint, liver, lung and eye diseases, the third covers mental and neurologic diseases and the fourth pattern includes gastrointestinal diseases and cancer. 44% of the persons were assigned to at least one of the four multimorbidity patterns; 14% could be assigned to both the cardiovascular/metabolic and the joint/liver/lung/eye pattern. Further common pairs were the mental/neurologic pattern combined with the cardiovascular/metabolic pattern (7.2%) or the joint/liver/lung/eye pattern (5.3%), respectively. Our results confirmed the existence of co-occurrence of certain diseases in elderly persons, which is not caused by chance. Some of the identified patterns of multimorbidity and their overlap may indicate common underlying pathological mechanisms.

## Introduction

Multimorbidity is commonly defined as coexistence of two or more chronic diseases in the same individual [1]. It is associated with reduced health outcomes including functioning and quality of life [2], [3], more complex clinical management [4], specific health care needs [5], [6] and increased health care costs [7].

Since chronic diseases are associated with advanced age and the number of aged persons in the population is rising, multimorbidity increasingly becomes an important issue in health care. Studies indicate that older people are more prone to develop two or more chronic conditions than younger ones [8], [9]. Thus, many studies have been limited in focus to older persons when studying multimorbidity. Reported prevalence rates of multimorbidity in aged populations vary considerably depending on data sources, considered diagnoses and study populations [10]. In Germany, the prevalence of multimorbidity ranged from 67.3% in the 50–75 year old population studied by Nagel et al. [11] to 73% in the study of Van den Bussche et al. [12] who analyzed claims data of 123,224 insured policy holders aged 65 and older. A recent systematic review of 41 published studies worldwide reported a range of multimorbidity between 55 and 98% of persons aged 65 and older [13].

Diseases that are common in older people may occur together by chance. However, often “common pathways” may lead to clustering of major chronic conditions. The impact of co-occurrence on functional status is sometimes greater than the sum of the impacts of the individual diseases [14]. Thus, analysis and exploration of “common pathways” of these co-occurring conditions offers the potential for improved medical management and for targeted interventions.

However, only few studies have explored the natural clustering of chronic conditions in aged populations so far. Some studies identified the most common comorbid pairs [14]–[16] or analyzed triadic combinations of diseases [12]. Only a few studies used cluster analysis in order to obtain a general picture of the broad pattern of how diseases are associated in a particular population [16]–[18]. Schäfer et al. [19] were the first who applied factor analysis as a method to explore multimorbidity patterns and identified three patterns: cardiovascular/metabolic disorders, anxiety/depression/somatoform disorders and pain, and neuropsychiatric disorders.

The objective of the present study was to explore patterns of comorbidity and multimorbidity in a Southern German population aged 65–94 years.

## Methods

### Ethics statement

The KORA-Age study was approved by the Ethics Committee of the Bavarian Medical Association. Written informed consent has been obtained from the participants and all investigations have been conducted according to the principles expressed in the Declaration of Helsinki.

### Study population

Data from the present study derived from the “KORA-Age” study which is a follow-up of all individuals aged 65–94 who have participated in at least one of the four cross-sectional MONICA/KORA surveys. These surveys have been conducted between 1984 and 2001 and included a random sample of the population of the city of Augsburg and its two surrounding counties in Southern Germany [20]. Details about study design, sampling method and data collection are reported elsewhere [21]. In total, 17,607 persons participated in at least one of the four surveys. The KORA-Age study population is restricted to the subgroup of 9,197 subjects who were born in 1943 or earlier. 2,734 of these 9,197 individuals died, 45 moved abroad or to an unknown location, and 427 refused to be contacted for any follow-up. A brief self-administered questionnaire was mailed to the entire KORA sample including the remaining 5,991 eligible persons of the KORA-Age subgroup with known address between November 2008 and September 2009. All persons who did not answer within 4 weeks were sent a postcard reminder. Four weeks later, non-responders were contacted by telephone and if the persons agreed to participate, the questionnaire was administered via telephone. In total, questionnaire data could be collected for 4,565 persons (response 76.2%), of whom 3,833 returned the questionnaire by mail and 732 (16.0%) were interviewed via telephone.

In addition to the data collected by questionnaire, in order to collect more specific data relevant for elderly people, an extended telephone interview was conducted with the 5,986 eligible persons with known address between December 2008 and November 2009. Of these, 1,859 persons could not be contacted or refused to participate. For 4,127 eligible persons, a telephone interview could be performed either with the persons themselves (n = 3,942) or with a proxy (i.e. a family member or care giver) (n = 185).

### Data collection

The presence of chronic health conditions was self-reported by the participants in the self-administered questionnaire and the standardized telephone interview. In the questionnaire the participants were asked whether they were ever diagnosed with a myocardial infarction, hypertension, stroke, diabetes mellitus or a malignant disease. In addition, participants should indicate whether they had a coronary artery bypass graft surgery or received a coronary artery stent.

The telephone interview was based on the self-report-generated Charlson Comorbidity Index [22] (see Table 1). The participants were requested to indicate whether they currently have asthma, emphysema or chronic obstructive pulmonary disease, an inflammatory joint disease (e.g. arthritis) or a rheumatic disease, a gastrointestinal disease (e.g. gastric or duodenal ulcer, colitis, cholecystitis), heart complaints (e.g. angina pectoris, heart failure, coronary heart disease), a kidney disease, or a liver disease (e.g. cirrhosis). In addition to the disorders covered by the Charlson Comorbidity Index, we asked for the presence of neurologic diseases such as multiple sclerosis, Parkinson's disease, or epilepsy, whether the participant has or had a glaucoma or cataract, and finally we requested the presence of any other disease which has not been mentioned before. Depression was assessed using the Geriatric Depression Scale (GDS-15) [23]. Scores above 10 points were considered framing depression. The presence of an anxiety disorder was assessed using the Generalized Anxiety Disorder Scale-7 (GAD) [24]. Scores ≥10 points indicated the presence of an anxiety disorder. An eye disease was deemed to be present if participants indicated to have glaucoma or cataract or other eye diseases such as macular degeneration, diabetic retinopathy, and retinitis pigmentosa. Each single response to the open question on other diseases was checked. If they contained information regarding the above mentioned conditions, the presence of the condition was modified accordingly. According to the available information on diseases, 13 conditions were defined (Table 2). Multimorbidity was defined as the co-occurrence of two or more of these conditions within one person.

**Table 1:** Assessment of diseases in the KORA-Age Study.

Self-report generated Charlson Comorbidity Index	KORA-Age Study	
Disease	Disease	Source
Asthma, emphysema, or chronic bronchitis	✓	Telephone interview
Arthritis or rheumatism	✓	Telephone interview
Cancer, diagnosed in the past 3 years	✓	Questionnaire
Diabetes	✓	Questionnaire
Digestive problems (such as ulcer, colitis, or gallbladder disease)	✓	Telephone interview
Heart trouble (such as angina, congestive heart failure, or coronary artery disease)	✓	Questionnaire, telephone interview
Kidney disease	✓	Telephone interview
Liver problems (such as cirrhosis)	✓	Telephone interview
Stroke	✓	Questionnaire
HIV illness or AIDS	-	Not requested*
	Neurologic diseases such as multiple sclerosis, Parkinson's disease, or epilepsy	Telephone interview
	Eye diseases such as glaucoma, cataract, macular degeneration, diabetic retnopathy, or retinitis pigmentosa	Telephone interview
	Hypertension	Questionnaire
	Depression	Geriatric Depression scale (GDS-15) administered via telephone interview
	Anxiety	Generalized Anxiety Disorder Scale-7 (GAD) administered via telephone interview

*none of the participants reported antiretroviral therapy in the medication history.

**Table 2:** Prevalences of the 13 chronic conditions.

Condition	Prevalence [%]	95% confidence interval
Hypertension	57.9	[56.3–59.4]
Eye disease	38.1	[36.6–39.6]
Heart disease	25.8	[24.4–27.1]
Diabetes mellitus	16.8	[15.6–17.9]
Joint disease	16.2	[15.1–17.4]
Lung disease	10.3	[9.4–11.2]
Gastrointestinal disease	8.7	[7.8–9.6]
Mental disease	8.4	[7.5–9.3]
Stroke	7.0	[6.2–7.8]
Cancer	4.3	[3.6–4.9]
Kidney disease	4.0	[3.4–4.6]
Neurological disease	3.4	[2.9–4.0]
Liver disease	2.4	[1.9–2.9]

### Data analysis

Age-standardized prevalences were calculated for each disease and the presence of multimorbidity using the age distribution of the German population on December 31, 2007. Occurrence of multimorbidity was estimated by logistic regression modelling adjusting for age and sex. The frequency of disease pairs was computed. For the disease pairs with a frequency >5%, the expected prevalence (prevalence disease A)×(prevalence disease B) was computed and compared with the observed prevalence. In order to determine the strength of the association between each pair of co-occuring diseases and to control for possible confounders, logistic regression with adjustment for age, sex, and all other diseases was performed. The level of significance was set to p<0.0042 applying Bonferroni correction for multiple testing.

Patterns of multimorbidity were analyzed by exploratory factor analysis. We applied the principal factor method and used a tetrachoric correlation matrix, which takes into account the dichotomy of the data [25]. The Kaiser-Meyer-Olkin measure was calculated to estimate the sampling adequacy for performing a factor analysis, and cumulative percent described the proportion of variance of the morbidity data that can be explained by the patterns. Factors with an eigenvalue of at least 1.0 were considered as substantial. We assumed that there will be associations among the factors, thus we used an oblique (oblimin) rotation of factor loading matrices.

We examined the frequencies of multimorbidity patterns by assigning individuals to a pattern if they experienced at least two diseases with a factor loading of ≥0.25 on the corresponding pattern. Age and sex-related differences in the pattern prevalence were analyzed using logistic regression modelling.

## Results

Of the 4127 subjects, 51.2% were female. 66.2% were married or lived with a partner. The mean age was 73.4±6.1 years. Women were significantly less likely to be married or to live with a partner than men (52.2% vs. 80.8%). Hypertension, eye diseases, and heart diseases were the most common disorders, with prevalences of 57.9%, 38.1%, and 25.8%, respectively (Table 2). The number of disorders ranged from 0 to 10 (Table 3). The median number of conditions was 2 (25^th^ percentile: 1; 75^th^ percentile: 3). Women did not differ significantly from men, whereas a higher number of conditions was related to older age.

**Table 3:** Number of conditions stratified by age group and sex.

No. of conditions	Total sample (n = 4067)	<75 years (n = 2541)		75–84 years (n = 1315)		≥85 years (n = 211)	
		Men (n = 1225)	Women (n = 1316)	Men (n = 664)	Women (n = 651)	Men (n = 93)	Women (n = 118)
0	507 (12.5%)	211 (17.2%)	197 (15.0%)	57 (8.6%)	33 (5.1%)	3 (3.2%)	6 (5.1%)
1	1096 (27.0%)	370 (30.2%)	416 (31.6%)	142 (21.4%)	141 (21.7%)	19 (20.4%)	8 (6.8%)
2	1093 (26.9%)	335 (27.3%)	357 (27.1%)	161 (24.3%)	186 (28.6%)	21 (22.6%)	33 (28.0%)
3	713 (17.5%)	187 (15.3%)	193 (14.7%)	149 (22.4%)	129 (19.7%)	20 (21.5%)	35 (29.6%)
4–6	621 (15.3%)	116 (9.5%)	142 (10.8%)	147 (22.1%)	156 (24.0%)	28 (30.1%)	32 (27.1%)
>6	37 (0.8%)	6 (0.5%)	11 (0.8%)	8 (1.2%)	6 (0.9%)	2 (2.2%)	4 (3.4%)
≥2	2464 (60.6%)	581 (47.4%)	613 (46.6%)	465 (70.0%)	477 (73.3%)	71 (76.3%)	104 (88.1%)

The prevalence of multimorbidity was 58.6% (95% confidence interval: 57.0–60.2) in the total sample. Logistic regression analysis revealed a significant interaction effect of age and sex regarding the occurrence of multimorbidity (p = 0.02). While multimorbidity was significantly associated with increasing age, only in the age group ≥85 years women were significantly more likely than men to have ≥2 conditions.

Table 4 shows the 12 most common pairs of conditions, their observed and expected frequency and the odds ratios testing their association. Hypertension, eye diseases, diabetes mellitus, and heart diseases were most often associated with a second condition. The occurrence of hypertension in combination with heart disease, diabetes, or stroke, respectively was more likely than expected, as well as the occurrence of eye diseases in combination with diabetes, joint diseases or lung diseases. Heart diseases also occurred more often than expected in combination with diabetes or joint diseases. These results were confirmed after adjustments for age, sex, and all other diseases (Table 4). Hypertension and diabetes, as well as hypertension and stroke emerged as the most strongly associated pairs of diseases.

**Table 4:** Most frequently co-occuring pairs of conditions and their observed and expected prevalences.

	Observed	Expected	Ratio observed/expected	Crude OR [99.58% CI]	p-value	Adjusted OR [99.58% CI]*	p-value^#^
Hypertension and eye diseases	23.80	22.64	1.05	1.21 [1.01–1.46]	0.003	1.04 [0.84–1.28]	0.632
Hypertension and heart diseases	18.39	15.58	1.18	1.86 [1.50–2.30]	<.001	1.67 [1.32–2.11]	<.001
Hypertension and diabetes	13.68	10.11	1.35	3.20 [2.41–4.25]	<.001	2.95 [2.19–3.96]	<.001
Hypertension and joint diseases	10.08	9.70	1.04	1.13 [0.88–1.44]	0.160	1.00 [0.77–1.30]	0.992
Eye diseases and diabetes	8.11	6.62	1.22	1.53 [1.20–1.94]	<.001	1.36 [1.04–1.77]	0.001
Eye and joint diseases	7.57	6.35	1.19	1.45 [1.14–1.85]	<.001	1.25 [0.96–1.63]	0.018
Hypertension and lung diseases	6.78	6.23	1.09	1.28 [0.95–1.73]	0.020	1.13 [0.82–1.57]	0.268
Heart diseases and diabetes	6.39	4.56	1.40	1.84 [1.43–2.36]	<.001	1.46 [1.11–1.92]	<.001
Eye and lung diseases	5.43	4.08	1.33	1.81 [1.35–2.42]	<.001	1.65 [1.20–2.28]	<.001
Hypertension and stroke	5.32	4.12	1.29	2.39 [1.59–3.59]	<.001	2.00 [1.26–3.16]	<.001
Heart diseases and joint diseases	5.31	4.37	1.21	1.40 [1.08–1.82]	0.002	1.24 [0.93–1.64]	0.034
Hypertension and gastrointestinal diseases	5.30	4.70	1.13	1.07 [0.77–1.47]	0.562	0.93 [0.66–1.31]	0.530

Results from logistic regression models testing the association between pairs of conditions.

*Odds ratios (OR) were adjusted for age, sex and all other diseases.

In the factor analysis, four factors emerged with a total of 47.8 cumulative percent. The Kaiser-Meyer-Olkin measure of 0.72 indicated a moderate sampling adequacy. The first pattern (eigenvalue 1.90) was characterized by high loadings for hypertension, heart diseases, diabetes and stroke and could be named as cardiovascular/metabolic disorders. The second factor (eigenvalue 1.76) was characterized by moderate factor loadings of joint, liver, lung and eye diseases. The third factor (eigenvalue 1.61) could be characterized by high factor loadings of mental diseases and neurologic diseases, and consequently was interpreted as multimorbidity pattern of mental and neurologic disorders. The fourth pattern (eigenvalue 1.54) showed high factor loadings of gastrointestinal diseases and cancer. While some diseases including hypertension, mental disorders and cancer were clearly associated with only one factor, six out of the 13 disorders showed factor loadings >0.25 on at least two factors.

Table 5 depicts the frequencies of the four patterns. The cardiovascular/metabolic diseases pattern was the most common one, followed by the joint/liver/lung and eye diseases pattern. The association of the occurrence of the single patterns with age and sex was analyzed using logistic regression modelling. Higher age was significantly related to a higher frequency of joint, liver, lung and eye diseases after adjustments for sex and the interaction between age and sex (p = .001; odds ratio (OR) 1.068, 95% confidence interval [1.029–1.108]. Regarding the occurrence of the cardiovascular/metabolic disease pattern, a significant interaction effect between age and sex (p = .006) was found with a higher occurrence for males in the younger age groups and a lower in the oldest age group. Regarding the two further patterns, no significant age or sex differences were found.

**Table 5:** Number and percent of persons who could be assigned to the multimorbidity patterns.

	Total (n = 2846)	Men (n = 1411)	Women (n = 1435)	p-value	<75 years (n = 1439)	75–84 years (n = 1174)	≥85 years (n = 233)	p-value
Cardiovascular/metabolic disorders	1314 (31.8%)	699 (34.7%)	615 (29.1%)	<0.01	678 (47.1%)	539 (45.9%)	97 (41.6%)	<0.01
Liver/lung/joint/eye disorders	1041(25.2%)	479 (23.7%)	562 (26.7%)	0.04	490 (34.1%)	456 (38.8%)	95 (40.8%)	<0.01
Mental/neurologic disorders	398 (9.7%)	187 (9.3%)	211 (10.0%)	0.44	219 (15.2%)	150 (12.8%)	29 (12.4%)	<0.01
Gastrointestinal disorders and cancer	93 (2.3%)	46 (2.3%)	47 (2.2%)	0.90	52 (3.6%)	29 (2.5%)	12 (5.2%)	0.01

Results of Chi^2^-test for sex and age-group differences.

Among the total sample, 56.0% could not be assigned to any of the four disease patterns. 24.8% of the people could be assigned to one disease pattern, 14.4% to two, 4.2% to three, and 0.7% to all four patterns.

When analyzing pair-wise occurrence of disease pattern, 14% of the people could be assigned to both the cardiovascular/metabolic pattern and the joint/liver/lung/eye pattern. Further common pairs are the mental/neurologic pattern combined with the cardiovascular/metabolic pattern (7.2%) or the joint/liver/lung/eye pattern (5.3%).

## Discussion

In the present study we used different approaches to describe comorbidity and multimorbidity in the elderly German population. Hypertension and diabetes, as well as hypertension and stroke were the two diseases which occurred most commonly in combination. This association was independent of age, sex and the presence of other conditions. Furthermore, we identified four patterns of multimorbidity: the first pattern includes cardiovascular and metabolic diseases, the second includes joint, liver, lung and eye diseases, the third covers mental and neurologic diseases and the fourth pattern includes gastrointestinal diseases and cancer.

In general, it is difficult to compare results across different studies, because these remarkably differ regarding data sources, populations, and number and type of diseases [10]. However, the prevalence of multimorbidity of 58.6% in our study fits well with the reported range of multimorbidity rates in elderly populations [3], [9], [11], [13], [26]. While the relation of multimorbidity with age is well documented [8], [26], [27], the reports on differences between the sexes are conflicting. Some studies did not retrieve significant differences between women and men regarding multimorbidity [8], [12], [27] whereas others found women older than 65 [26] or 77 years [28] to be more likely to be multimorbid than men. In our study, only women aged ≥85 years were more likely than men to report two or more conditions.

The most common comorbid pairs found in our study were hypertension and diabetes, hypertension and stroke, as well as hypertension and heart disease which reflect well-established pathophysiological associations [29]–[31]. Furthermore, the association between heart disease and diabetes indicated the existence of a cardiovascular/metabolic disorder pattern. Consistent with the pair-wise associations between cardiovascular disorders and diabetes, the factor analysis identified a pattern that covered these conditions and had the highest frequency of 31.8% within the study sample.

Regarding the pair-wise co-occurrence of diseases, we found a significant association between eye diseases and diabetes. It may be explained by the possible microvascular complications of diabetes such as diabetic retinopathy [32], and the occurrence of cataract, which was reported to be more common in persons with diabetes than in non-diabetic persons [33]. Furthermore, rheumatoid arthritis can have a number of extraarticular manifestations related to the heart as well as to the eyes [34]. This fact might have contributed to the significant relation between joint diseases and heart diseases, and joint diseases and eye diseases, respectively, detected in our study. Finally, eye and lung diseases co-occurred significantly more often than expected. This finding contributes to the current controversial discussion about a relation between the systemic inflammation and/or decreased systemic oxygenation associated with lung diseases and the risk of age-related macular degeneration [35], [36]. An additional potential explanation for this finding could be the reported increased risk of cataracts associated with long-term use of corticosteroids in the treatment of asthma [37].

In accordance with other studies which examined multimorbidity using cluster or factor analysis, we found a pattern of cardiovascular and related diseases. In two other studies, metabolic diseases [18] or stroke [17] were included in this pattern. We can confirm the results of Schäfer et al. [19] that both conditions, diabetes and stroke, were assigned to the cardiovascular/metabolic pattern.

Moreover, we found a disease pattern that covers mental disorders (depression, anxiety) and neurologic disorders such as Parkinson's disease, multiple sclerosis or epilepsy. This finding is overall consistent with Cornell et al. [18] who identified a mixed anxiety-depression cluster in their sample. Neurologic disorders like Parkinson's disease, multiple sclerosis or epilepsy were not considered in their study. Our results are consistent with the growing literature on the comorbid relationship and biological association between depression and anxiety [38], [39] as well as on the frequent association of depression and anxiety with other neurologic diseases [40], [41].

Of interest, we demonstrated in our study that the mental/neurologic pattern frequently overlapped with the cardiovascular/metabolic pattern or the joint/liver/lung/eye pattern. This finding supports the current discussion about inflammatory as well as oxidative and nitrosative stress pathways that underpin the common pathophysiology of depression and disorders such as cardiovascular disease, diabetes, stroke, chronic obstructive pulmonary disease, rheumatoid arthritis, and inflammatory bowel disease, and that activation of these pathways contributes to shared risk [42], [43].

Since the mental/neurologic pattern emerged as a relevant factor of multimorbidity in our study, we agree with Maes et al. [42] that mental conditions should be assessed in studies on multimorbidity in the elderly population. It may also be useful to include dementia, which was not done in our study. Unfortunately, neither mental nor neurological conditions are included in the most frequently used comorbidity measure, the Charlson Comorbidity Index [22].

Our study is the second published study which applied factor analysis methodology to explore the structure of multimorbidity in elderly people. Recently, Schäfer et al. [19] have demonstrated the benefit of applying a factor analytical approach which allows diseases to be associated with more than one pattern, in contrast to cluster analysis. They have shown that seven out of 46 diseases included in their analysis where associated with different patterns, for instance depression, stroke and cardiac insufficiency. In our study we could confirm that almost one half of the 13 diseases, including stroke and heart diseases, correlated with more than one pattern, whereas the mental diseases including depression were clearly associated with only one factor. In accordance with Schäfer et al. [19], we detected a limited number of factors that could be well interpreted. The model fit, however, was moderate with a rate of cumulative percent of 48% and a Kaiser-Meyer-Olkin measure of 0.72.

The number and selection of diseases considered in our study and the self-report methodology used, surely will have affected our results. Although our assessment of diseases was generally based on the Charlson Comorbidity Index [22], we have added some disorders which seemed highly relevant for exploring multimorbidity in the elderly population, such as hypertension, eye diseases, mental diseases and neurological diseases. On the one hand, this approach has reduced standardization and comparability with other studies. On the other hand, these additional disorders emerged as the most important ones in the analysis and thus contributed to the validity of the study. Furthermore, we used standard multi-item scales for the assessment of depression and anxiety as compared to simply ask patients for the presence of these problems. This might also have influenced our results. In addition, we have only assessed the occurrence of a disease without taking into account the severity. Finally, our study had a cross-sectional design and therefore allows no conclusions regarding causal relationships among disease clusters.

Underlying these limitations is the lack of an internationally accepted standard for assessing multimorbidity. A conceptual framework including a consistent measurement approach of multimorbidity for different study populations would strongly facilitate comparisons between studies and populations [27].

The results of our study contribute to a deeper understanding of the complexity of multimorbidity by characterizing the association between diseases in multiple ways. Further research is needed to determine the impact of different multimorbidity patterns on various health outcomes including mortality, disability, quality of life, and health care costs. Negative synergy effects of multiple disease patterns in individual patients need further attention [3]. Specifically, the knowledge about the health care needs associated with different multimorbidity patterns might help us to improve the life of elderly persons with multiple diseases.
